# A preliminary study on improving the recognition of esophageal speech using a hybrid system based on statistical voice conversion

**DOI:** 10.1186/s40064-015-1428-2

**Published:** 2015-10-26

**Authors:** Othman Lachhab, Joseph Di Martino, Elhassane Ibn Elhaj, Ahmed Hammouch

**Affiliations:** LRGE Laboratory, ENSET, Mohammed 5 University, Madinat Al Irfane, Rabat, Morocco; LORIA, B.P. 239, Vandœuvre-lès-Nancy, 54506 France; INPT, Madinat Al Irfane, Rabat, Morocco

**Keywords:** Speech enhancement, Esophageal speech assessment, Voice conversion, Pathological voices, Automatic speech recognition (ASR)

## Abstract

In this paper, we propose a hybrid system based on a modified statistical GMM voice conversion algorithm for improving the recognition of esophageal speech. This hybrid system aims to compensate for the distorted information present in the esophageal acoustic features by using a voice conversion method. The esophageal speech is converted into a “target” laryngeal speech using an iterative statistical estimation of a transformation function. We did not apply a speech synthesizer for reconstructing the converted speech signal, given that the converted Mel cepstral vectors are used directly as input of our speech recognition system. Furthermore the feature vectors are linearly transformed by the HLDA (heteroscedastic linear discriminant analysis) method to reduce their size in a smaller space having good discriminative properties. The experimental results demonstrate that our proposed system provides an improvement of the phone recognition accuracy with an absolute increase of 3.40 % when compared with the phone recognition accuracy obtained with neither HLDA nor voice conversion.

## Background

A total laryngectomy is a surgical procedure which consists in a complete removal of the larynx for the treatment of a cancer for example. Thus, the patient loses his/her vocal cords that allowed him/her a laryngeal voice. After surgery, some patients may waive any oral communication attempt because of the physical and mental bouleversement caused by the surgical act. Indeed, the anatomical changes deprive temporarily the patient of his/her voice. Only the whispered voice allows communication in a postoperative life. An alternative speaking rehabilitation method allows him/her to get a new voice called esophageal speech (ES) generated without vocal folds. The air from the lungs, original source of all human speech, no longer passes through the cavities of the phonatory apparatus. It is released directly from the stomach through the esophagus. The features of esophageal speech such as the envelope of the waveform and the spectral components differ from the features extracted from natural speech. Furthermore, the esophageal speech is characterized by specific noises and low intelligibility; the fundamental frequency of this voice is less stable than that of laryngeal voice. All these aspects cause a production of a hoarse, creaky and unnatural voice, difficult to understand.

Currently, researchers are mostly concentrated on the recognition and evaluation of alaryngeal speech, in such fields as laryngology and biomedical application of speech technology (Pravena et al. 2012; Dibazar et al. 2006). The evaluation of esophageal speech by perception judgments is one of the most used methods in clinical practice. It consists in following postoperative vocal evolution and efficiency of reeducation. The major drawbacks of this approach are the missing of reliability, as well as the difficulty of establishing a jury of experts for listening. Given the limitations of this perceptual analysis, the establishment of a more objective assessment protocol becomes a necessity. Nowadays, instrumental analysis (Wuyts et al. 2000; Yu et al. 2001) aims to provide a solution based on acoustic and aerodynamic measurements of speech sounds. Recently in (Lachhab et al. 2014), we proposed a new objective technique to assess esophageal speech. The originality of this approach is based on the use of an automatic speech recognition system in order to extract phonetic information of pathological voice signals.

In this paper, we propose a new hybrid system based on statistical voice conversion for improving the recognition of esophageal speech. This enhancing system combines a voice conversion algorithm that transforms esophageal speech into a “target” laryngeal speech, with an automatic speech recognition system based on HMM^1^/GMM^2^ models. This approach aims to correct and extract the lexical information contained in esophageal speech. Our hybrid system does not apply a speech synthesizer for reconstructing the converted speech signal, because the automatic speech recognition system used needs only as input data, converted Mel cepstral features. The discriminant information of the converted acoustic vectors is increased by the HLDA (heteroscedastic linear discriminant analysis) transformation in order to improve system performance.

This paper is organized as follows: “Previous and current research on enhancing pathological speech” details previous and current works on enhancing pathological voice. The used corpora for voice conversion and the HLDA transformation method are described in “The FPSD corpus” and “The HLDA transformation” respectively. In “The hybrid system for enhancing esophageal speech”, the proposed hybrid system for improving the recognition of esophageal speech is discussed. In “Experiments and results”, we present the experiments and obtained results. Finally, a conclusion of this paper is provided in “Conclusion and future works” as well a list of possible future works.

## Previous and current research on enhancing pathological speech

The esophageal speech is characterized by high noise perturbation, low intelligibility and a fundamental frequency which is unstable. All these characteristics when compared with those of the laryngeal speech produce a hoarse, creaky and unnatural voice, difficult to understand. For this reason, several approaches have been proposed to improve the quality and intelligibility of the alaryngeal speech. One such a method described in (Qi et al. 1995), consists in resynthesizing tracheoesophageal (TE) speech using a simulated glottal waveform and a smoothed F0. A similar approach (del Pozo and Young 2006), uses a synthetic glottal waveform and a jitter and shimmer reduction model to reduce breathiness and harshness of original TE speech. Some other authors have proposed a signal processing based speech prosthesis, such Mixed-Excitation Linear Prediction (MELP) (Türkmen and Karsligil 2008), which consists in synthesizing normal speech from whispered voice by using pitch estimation and formant structure modification on voiced phonemes. The unvoiced phonemes in this study remain unmodified. However, this technique is unsuited to real-time operation. Another exemple has been reported by (Sharifzadeh et al. 2010), with a Code-Excitation Linear Prediction (CELP) in order to produce more natural characteristics by reconstructing the missing pitch elements from whispered speech. However, it is still difficult to mechanically generate realistic excitation signals similar to the one naturally generated by vocal fold vibrations. Other attempts for enhancing pathological speech based on the modifications of their acoustic features have been proposed, such as formant synthesis (Matui et al. 1999), background noise reduction based on auditory masking (Liu et al. 2006), approximation of vocal tract using LPC (Garcia et al. 2002, 2005) and comb filtering (Hisada and Sawada 2002), denoising electrolarynx (EL) speech by combined spectral substraction and root cepstral substraction procedure (Cole et al. 1997). This subtractive-type method is limited and lacks of accuracy in estimation of the background noise. In (Mantilla-Caeiros et al. 2010), the esophageal speech enhancement system proposed aims to replace voiced segments of alaryngeal speech, selected by pattern recognition techniques, with corresponding segments of normal speech. The silence and unvoiced segments remain unchanged. Another work reported in (del Pozo and Young 2008), consists in repairing TE phone durations by those predicted by regression trees built from normal data.

Recently, a statistical approach for enhancing alaryngeal speech based on conversion voice has been proposed in (Doi et al. 2014). This technique consists in converting the alaryngeal speech sound, in order to be perceived as pronounced by a target speaker with a laryngeal voice. In (Tanaka et al. 2014), a new hybrid method for alaryngeal speech enhancement based on noise reduction by spectral subtraction (Boll 1979) and using statistical voice conversion for predicting the excitation parameters was developed. These two recent approaches aim to improve the estimation of acoustic features in order to reconstruct an enhanced signal with best intelligibility. However, the conversion process used in these methods is quite complex and can generate errors in parameters estimation and thus produce unnatural synthesized sounds due to the lack of realistic excitation signals related to the converted spectral parameters. Consequently, in practice it is difficult for them to compensate for the differences existing in the alaryngeal acoustic parameters when compared with those of the laryngeal speech.

To overcome this drawback, we propose a new hybrid system for improving the recognition of esophageal speech based on a simple voice conversion algorithm. In this conversion process, an iterative statistical estimation of a transformation function is used. This estimation method is computationally inexpensive when compared to the classical EM (Werghi et al. 2010). On the other hand, we do not use a synthesizer for reconstructing the converted speech signal, because our hybrid system integrates a speech recognition system in order to extract the phonetic information directly from converted MFCC*^3^ vectors.

## The FPSD corpus

We chose to develop our esophageal speech recognition system with our own database. This French database entitled FPSD (French Pathological Speech Database), was established to simplify the training of phonetic models of esophageal speech recognition systems. This corpus contains 480 audio files saved in wav format, accompanied with their orthographic transcription files. The sentences are pronounced by a single laryngectomee speaker. We organized all the data in packets of five categories:

1. Sentences with one-syllable words.
2. Sentences with words of one and two syllables.
3. Sentences with words of three syllables.
4. Sentences with falling intonation.
5. Sentences with rising intonation.

**Table 1:** SAMPA transcription of the standard French phones

Number	IPA	SAMPA	Example
1	p	p	*p*ont [po$\sim$]
2	b	b	*b*on [bo$\sim$]
3	t	t	*t*emps [ta$\sim$]
4	d	d	*d*ans [da$\sim$]
5	k	k	*c*oût [ku]
6	g	g	*g*ant [ga$\sim$]
7	f	f	*f*emme [fam]
8	v	v	*v*ent [va$\sim$]
9	s	s	*s*ans [sa$\sim$]
10	z	z	*z*one [zOn]
11	j	j	*i*on [jo$\sim$]
12	m	m	*m*ont [mo$\sim$]
13	n	n	*n*om [no$\sim$]
14		N	ri*ng* [riN]
15	ʃ	S	*ch*amp [Sa$\sim$]
16	ʒ	Z	*g*ens [Za$\sim$]
17	ɔ	O	c*o*mme [kOm]
18	o	o	gr*o*s [gRo]
19	u	u	d*ou*x [du]
20	y	y	d*u* [dy]
21	ə	@	d*e* [d@]
22	l	l	*l*ong [lo$\sim$]
23	ʁ	R	*r*ond [Ro$\sim$]
24	w	w	q*u*oi [kwa]
25	ɥ	H	j*u*in [ZHe$\sim$]
26	i	i	s*i* [si]
27	e	e	bl*é* [ble]
28	ɛ	E	s*ei*ze [sEz]
29	a	a	p*a*tte [pat]
30	ø	2	d*eu*x [d2]
31	œ	9	n*eu*f [n9f]
32		9$\sim$	br*un* [br9$\sim$]
33		e$\sim$	v*in* [ve$\sim$]
34		a$\sim$	v*en*t [va$\sim$]
35		o$\sim$	b*on* [bo$\sim$]
36	sil	- or sil	*silence*

It is necessary to have a fairly large training corpus in order to process the intra-speaker variability. The more important is the training data, the better are the obtained performances. We divided our corpus into two subsets: one for training and the other one for the test. The training subset contains 425 sentences and the test one contains 55 sentences. The structure of our FPSD corpus is similar to the one used in the TIMIT corpus (Garofolo et al. 1993). We have for each sentence, the French text stored in a file (.txt), the audio signal recorded in the (.wav) format and sampled at 16 KHz with 16 bits by sample with a single input channel, a file (.wrd ) containing the word transcription and a file (.phn) containing the manual segmentation into phonemes. For realizing this manual segmentation we used the Praat^4^ software which allows both transcriptions, annotations and analysis of the acoustic data. This software allows also viewing spectrograms and calculating prosodic parameters such as intensity, fundamental frequency, and other parameters such as energy and formants. Indeed, although it is difficult to assess the quality of a phonetic segmentation, there is a broad consensus on the fact that manual segmentation is more accurate than automatic segmentation. The phonetic labeling of the sentences was carried out with SAMPA^5^ (Speech Assessment Methods Phonetic Alphabet) characters. This labeling method offers the advantage of using only simple ASCII characters. With SAMPA there is up to two characters to represent a phoneme. There exists another standard phonetic transcription method called International Phonetic Alphabet (IPA). Unfortunately, in the IPA method each phoneme is represented by a symbol that may not be entered on a computer keyboard. Table  1 shows the list of the 36 French phonetic labels used in our own FPSD database, with the IPA correspondence and examples.

## The HLDA transformation

The goal of HLDA (Kumar and Andreou 1998) method consists in transforming the original data in a reduced dimension space while preserving discriminant information and the de-correlation of the different classes (phonemes). The n-dimensional feature vectors are projected into a space of $p \le n$ dimension. Mathematically, we can express this reduction by applying the following linear transformation function:

1 $$\begin{aligned} Y=\Theta X=\left[ \begin{array}{c} \Theta _{p} X_{n} \\ \Theta _{n-p} X_{n} \end{array} \right] = \left[ \begin{array}{c} Y_{p} \\ Y_{n-p} \end{array} \right] \end{aligned}$$

where $\Theta _{p}$ represents the *p* first rows of the transformation matrix and $\Theta _{n-p}$ represents the remaining $n-p$ rows. To obtain the transformed vectors $Y_{p}$, we multiply the transformation matrix $\Theta _{p}$ of dimension $(p\times n)$ by the input vector $X_{n}$. Heteroscedastic LDA (HLDA) is an extension of LDA (Haeb-Umbach and Ney 1998). LDA assumes that the mean is the discriminating factor and not the variance, because the class distributions are Gaussians with different means and common covariance (Homoscedasticity). Due to this homoscedasticity, LDA may provide unsatisfactory performances when the class distributions are heteroscedastic (unequal variances or covariances). In order to overcome this limitation, HLDA has been proposed for treating the heteroscedasticity property. Each class is modeled as a normal distribution of $x_{i}$ training vectors.

2 $$\begin{aligned} p(x_{i})=\frac{|\Theta |}{\sqrt{(2\pi )^{n}|\Sigma _{c(i)}|}} \exp \left(-\frac{1}{2}(\Theta x_{i}-\mu _{c(i)})^{T}\Sigma _{c(i)}^{-1} (\Theta x_{i}-\mu _{c(i)})\right) \end{aligned}$$

where $\mu _{c(i)} , \Sigma _{c(i)}$ represent the mean vector and covariance matrix of class *c*(*i*) respectively. The objective is to find the optimal solution that respects a maximization criterion of log-likelihood probability function of the data in terms of $\Theta$.

3 $$\begin{aligned} \tilde{\Theta }=\arg \max _\Theta \sum _{\forall i}\log p(x_i) \end{aligned}$$

The efficient iterative algorithm based on the generalized Expectation Maximization (EM) proposed in (Gales 1999; Burget 2004), is used in our experiments to simplify the estimation of matrix $\Theta$.

## The hybrid system for enhancing esophageal speech

**Fig. 1:**
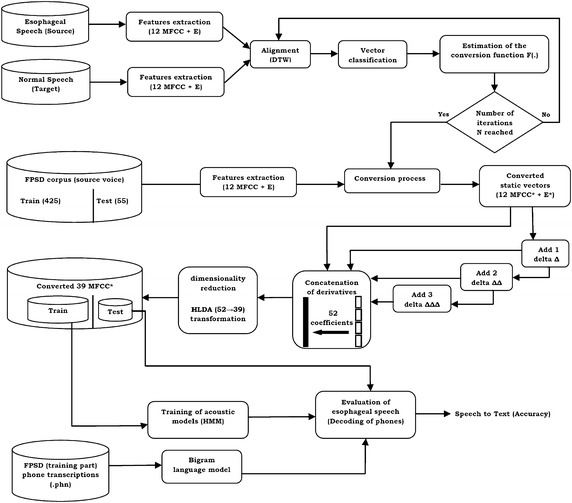
Block diagram of the proposed hybrid system for improving the recognition of esophageal speech

In this section, the theory and implementation of the hybrid system for esophageal speech enhancement are described in detail. A block diagram of the proposed system is shown in Fig. 1.

### Features extraction

The speech signals of the source and target speakers undergo a parameterization phase. The objective of this phase is to extract MFCC (Davis 1980) cepstral vectors. In this processing, the speech signal is sampled at 16 Khz with pre-emphasis of 0.97. A Hamming window of 25 ms shifted every 10 ms is used for obtaining the short time sections from which the cepstral coefficients are extracted. The first 12 cepstral coefficients (c1–c12) obtained from a bank of 26 filters in a Mel frequency scale, are retained. The logarithm of the energy of the frame, normalized over the entire sentence is added to the 12 cepstral coefficients in order to form a vector of 13 static coefficients (12 MFCC+ E).

### Statistical voice conversion

The voice conversion process can be decomposed into two steps: training and transformation. During the training step, a parameterization phase (features extraction) is applied on two parallel corpora (source and target voices) containing sentences with the same phonetic content. The extracted cepstral vectors are used for determining an optimal conversion function that transforms the source vectors into target ones while minimizing the mean square error between the converted and target vectors. The second step is the transformation in which the system uses the previously learned conversion function for transforming the source speech signals in order to be perceived as pronounced by the target speaker.

The purpose of voice conversion is to convert the characteristics of a sound signal from a source speaker into the characteristics of a target speaker. In this paper, we will consider the GMM Gaussian mixture-based method described by Stylianou et al. (1998) and improved by Kain and Macon (1998), Toda et al. (2007) and then by Werghi et al. (2010). The Werghi’s algorithm has been used in this study as our basic voice conversion procedure.

1. Training process: The *X* (source) sentences are normalized in a first step in order to have the same length in samples of their corresponding *Y* (target) normal voice sentences (this process is realized by the free Unix “sox” software) and aligned in a second step by the Dynamic Time Warping (DTW) algorithm. This latest phase consists in mapping the source vectors with the target vectors in order to create a huge mapping list. The corresponding vectors are concatenated then jointly in a single vector $z=[x \ y]^T$ before classification. These extended vectors are classified using the “k-means” vector quantization algorithm (Kanungo et al. 2000) in order to determine the initial GMM parameters. The joint probability of vector *z* is given by:
  4 $$\begin{aligned} p(z)=\sum _{i=1}^G\alpha _i\mathcal {N}_i(z,\mu _i,\Sigma _i) \end{aligned}$$
  $$\begin{aligned} \Sigma _i=\left[ \begin{array}{cc} \Sigma _{i}^{xx} &{} \Sigma _{i}^{xy} \\ \Sigma _{i}^{yx} &{} \Sigma _{i}^{yy} \end{array} \right] \ \ \ and \ \ \ \mu _i=\left[ \begin{array}{c} \mu _{i}^{x} \\ \mu _{i}^{y} \end{array} \right] \end{aligned}$$
  where $\mathcal {N}(\cdot ,\mu ,\Sigma )$ denotes a Gaussian distribution with a mean vector $\mu$ and a covariance matrix $\Sigma$, $\alpha$ is the mixture weight. This combination is used to model a joint GMM that depends on the source and target parameters. We obtain all the parameters at once, the mean vectors source and target $(\mu ^x,\mu ^y)$, the source and target covariance matrices $(\Sigma ^{xx},\Sigma ^{yy})$ and the cross-covariance matrices $(\Sigma ^{xy},\Sigma ^{yx})$ for each class *i*. The parameters are estimated by the iterative algorithm ISE2D (Iterative Statistical Estimation Directly from Data) described in (Werghi et al. 2010). The conversion function *F*(*x*) is then defined as the expectation *E*[*y* / *x*]:
  5 $$\begin{aligned}&F(x)=E[y/x]=\sum _{i=1}^Gp(x/i)(\mu _i^y+\Sigma _i^{yx}{(\Sigma _i^{xx})}^{-1} (x-\mu _i^x))\end{aligned}$$
  6 $$\begin{aligned}&p(x/i)=\frac{\alpha _i\mathcal {N}(x,\mu _i^x,\Sigma _{i}^{xx})}{\sum _{j=1}^G\alpha _j\mathcal {N}(x,\mu _j^x,\Sigma _j^{xx})} \end{aligned}$$
  where *p*(*x*/*i*) represents the posterior probability that *x* is generated by the *ith* component and *G* is the number of Gaussians. The ISE2D method is computationally less expensive and gives better results than the classical EM method. This approach consists in estimating the GMM parameters directly from data by statistical computations shown below:
  The weight $\alpha _i$ of each normal distribution is estimated as the ratio between $N_{s,i}$ the number of source vectors of class *i* and $N_s$ the total number of source vectors.
    7 $$\begin{aligned} \alpha _i=\frac{N_{s,i}}{N_s} \end{aligned}$$
  The mean source vector $\mu ^x$ and mean target vector $\mu ^y$ are computed as follows:
    8 $$\begin{aligned} \mu _i^x=\frac{\sum _{k=1}^{N_{s,i}}x_i^k}{N_{s,i}} \ \ \ and \ \ \ \mu _i^y=\frac{\sum _{k=1}^{N_{t,i}}y_i^k}{N_{t,i}} \end{aligned}$$
    where $x^k$ , $y^k$ and $N_{t,i}$ represent the $kth$ source vector, the $kth$ target vector and the number of target vectors of class *i*.
2. Conversion process: Once the GMM parameters are calculated, the previously estimated conversion function is applied to all the vectors of the FPSD database for converting the 12 MFCC*+E*^6^ vectors $\hat{y}_k$ .
  9 $$\begin{aligned} \hat{y}_k=F(x_k) \end{aligned}$$
  (k represents the vector number)

We do not use a synthesizer to reconstruct the speech signal. The converted vectors are used directly as input data of our speech recognition system.

### Adding derivatives and reducing the dimensionality by HLDA

We have developed the same algorithm used in HTK for calculating the three derivatives. Let *C*(*t*) the cepstral coefficients of the converted frame at time t, then the corresponding delta coefficients $\Delta C(t)$ are calculated on an analysis window of five frames ($N_{\Delta }= 2$) by using the following formula:

10 $$\begin{aligned} \Delta C(t)= \frac{\sum _{i=1}^{N_{\Delta }}i(C_{t+i}-C_{t-i})}{2\sum _{i=1}^{N_{\Delta }}i^2} \end{aligned}$$

The same formula (10) is applied to the delta coefficients to obtain the acceleration $(\Delta \Delta )$ coefficients. Similarly the third differential coefficients are computed by applying Eq. 10 on the acceleration $(\Delta \Delta )$ coefficients. The derivatives of the energy are calculated also in the same way. As mentioned above in “Statistical voice conversion”, the conversion is applied on the 13 static coefficients MFCC $(12 MFCC + E)$. The differential coefficients of order 1, 2 and 3 called dynamic coefficients ($\Delta$, $\Delta \Delta$ and $\Delta \Delta \Delta$) are calculated from converted static coefficients and concatenated in the same space in order to increase the number of coefficients to $d=52$. In order to improve the discriminant information and reduce the space dimensionality, the HLDA transformation matrix has been estimated using the method described in “The HLDA transformation”. The new converted discriminant vectors contain 39 coefficients which represents the reference dimensionality used in most Automatic Speech Recognition systems (ASR).

### The training of esophageal speech recognition system

**Fig. 2:**
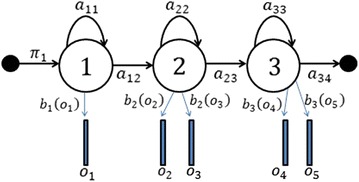
Topology of the context-independent phonetic HMM

Our esophageal speech recognition system is based on a statistical approach integrating acoustic and language levels in one decision process. These levels are represented by Hidden Markov Models (HMM). The 36 phones described in “The FPSD corpus” (see Table 1) are all modeled by left-to-right HMMs (see Fig. 2) with five states each (but only three of them can emit observations). The training of the acoustic models consists in estimating the mean vectors and covariance matrices of a set of weighted Gaussians. These parameters allow the computation of probability densities that constitute likelihood values associated with the emission of an observation by a state of a HMM. Furthermore the estimation of discrete probabilities associated with transitions between different states of the HMM are calculated. The converted discriminant vectors belonging to the training part of our FPSD database are used to estimate the optimal parameters $\{A, \pi _i, B\}$.

Where:

- $\pi _i$: An initial state probability.
- $A = a_{ij}$: The probability of transition from state *i* to state *j* (*A* is a transition probability matrix).
- $B = b_i(o_t)$: the matrix containing the distribution probability of emission the observation $o_t$ in state *i*.

The output distribution $b_i(o_t)$ for observing $o_t$ in state *i* is generated by a Gaussian Mixture Model (GMM) and more precisely by a mixture of multivariate Gaussian distribution probabilities $\mathcal {N}(o_t, \mu _{ik}, \Sigma _{ik})$ of mean vector $\mu _{ik}$ and covariance matrix $\Sigma _{ik}$:

11 $$\begin{aligned}&b_{i}(o_{t})= \sum _{k=1}^{n_{i}} \frac{c_{ik}}{\sqrt{{(2\pi )}^{d}|{\Sigma }_{ik}|}} \exp \left(-\frac{1}{2}(o_{t}-\mu _{ik})^{T}\Sigma ^{-1}_{ik}(o_{t}-\mu _{ik})\right)\nonumber \\&\left(\mathrm{with} \sum _{k=1}^{n_i}c_{ik}=1\right) \end{aligned}$$

where $n_i$ represents the number of Gaussians in state *i*, $o_t$ corresponds to an observation *o* at time *t* and $c_{ik}$ represents the mixture weight for the *kth* Gaussian in state *i*. The recognition system is implemented using the platform HTK (Young et al. 2006). The HMM parameters are estimated based on maximum likelihood criterion MLE (Rabiner 1989).The obtained models are improved by increasing the number of Gaussians used to estimate the probability of emission of an observation in a state. The choice of the optimal number of Gaussians is a delicate issue, generally guided by the amount of training data. In our case, we limited this number to 16 Gaussians by state.

### Phone recognition

The phone decoding is the heart of speech recognition systems. Its goal is to find the most likely states sequence corresponding to the parameters observed, in a composite model, and deducing the corresponding acoustic units. This task is performed using the Viterbi decoding algorithm applied on the converted Test vectors using the optimal parameters $\{A,\pi _i, B\}$ already estimated. In parallel of this alignment, a bigram language model is calculated on all of the training part of our FPSD database to improve the decoding. The bigram language can be represented by a two-dimensional table giving the probability of occurrence of two successive phonemes. In this study the bigram language has been trained using only 425 sentences from HTK modules. The inclusion of this model allows approximately a 10 % gain in accuracy. Our language model can be of course enriched by various textual contents of large French databases in order to improve the performances of our system.

## Experiments and results

In order to convert esophageal speech into a “normal speech” we recorded 50 esophageal and laryngeal sentences uttered respectively by a French male laryngectomee (the same one who participated in the creation of the FPSD database) and a French male speaker having a non-pathological voice. These new recordings do not belong to the FPSD database. They were uttered in order to determine the statistical conversion function. During the first iteration of training, the DTW alignment is applied on the source vectors *x* and target *y* containing 13 static coefficients. From the second iteration, the DTW alignment is realized between the converted static vectors $$\hat{y}$$ and target vectors *y* in order to refine the mapping list. The conversion function is estimated using 64 classes. For evaluating our hybrid system we performed three experiments on the phone recognition system level (the conversion experiment described previously does not change). In the first experiment, we computed the derivatives of order 1 and 2 from the converted static vectors using the same HTK regression formula. The purpose of this experiment is to recover dynamic information and have new dimension vectors $$= 39 \ (12$$$$MFCC^*, E^* ; 12$$$$\Delta MFCC^*, \Delta E^*; 12$$$$\Delta \Delta MFCC^*, \Delta \Delta E^*)$$ representing the reference dimensionality in most ASR systems. In experiment 2, another derivative ($$\Delta \Delta \Delta$$) is added and concatenated in the vectors space in order to increase the number of coefficients at $$d = 52 \ (12$$$$MFCC^*, E^*; 12$$$$\Delta MFCC^*, \Delta E^*; 12$$$$\Delta \Delta MFCC^*, \Delta \Delta E^*; 12$$$$\Delta \Delta \Delta MFCC^*, \Delta \Delta \Delta E^*)$$. In experiment 3, the space of 52 coefficients used in experiment 2 is reduced to 39 coefficients using the HLDA $$(52\rightarrow 39)$$ transformation for improving the discriminant information and reducing the space dimensionality.

The phone accuracy and correct rates are calculated by Eq. 12, in order to evaluate our esophageal speech recognition system where *N* represents the total number of labels of the test utterances. The Substitution (*S*), Insertion (*I*) and Deletion (*D*) errors are computed by the DTW algorithm between the correct phone strings and the recognized phone strings.

12 $$\begin{aligned} Accuracy=\frac{N-(S+D+I)}{N}; \ Correct=\frac{N-(S+D)}{N} \end{aligned}$$

Table 2 shows the results of the three experiments described above on the converted MFCC* vectors of the Test part of our own FPSD database containing 55 sentences.

An additional evaluation with the same experiments has been performed on our phone recognition system using the original FPSD database (without vector conversion). We also realized these experiments on the laryngeal voice TIMIT database (Garofolo et al. 1993) with the same 39 phonetic classes as described by Lee and Hon (1989).

**Table 2:** Influence of the number of differential coefficients with the HLDA transformation on phone recognition rates on the converted $MFCC^*$ vectors of the Test part of FPSD database

36 monophone HMMs with 16 Gaussians per state $+$ Bigram	Accuracy (%)	Correct (%)
Exp 1 : $39 \ MFCC^*$ coefficients	63.48	68.58
Exp 2 : $52 \ MFCC^*$ coefficients	61.78	67.36
Exp 3 : $HLDA \ (52\rightarrow 39)$	65.29	69.85

**Table 3:** Influence of the number of differential coefficients with the HLDA transformation on phone recognition rates on the Test part of the original FPSD database (without vector conversion)

36 monophone HMMs with 16 Gaussians per state $+$ Bigram	Accuracy (%)	Correct (%)
Exp 1 : $39 \ MFCC$ coefficients	61.89	67.62
Exp 2 : $52 \ MFCC$ coefficients	58.49	65.29
Exp 3 : $HLDA \ (52\rightarrow 39)$	63.59	69.43

**Table 4:** Influence of the number of differential coefficients with the HLDA transformation on phone recognition rates on the core test of the TIMIT database

39 monophone HMMs with 16 Gaussians per state $+$ Bigram	Accuracy (%)	Correct (%)
Exp 1 : $39 \ MFCC$ coefficients	69.19	71.78
Exp 2 : $52 \ MFCC$ coefficients	67.96	71.38
Exp 3 : $HLDA \ (52\rightarrow 39)$	71.32	74.07

The two tables, Tables 3 and 4 present the accuracy and correct rates for the three experiments described above respectively on the Test part of the original FPSD database (without vector conversion), and on the Core Test of the TIMIT database. From the results of experiment 3 (in Table 2) we can observe that the proposed hybrid system provides an improvement in phone recognition accuracy with an absolute increase of 3.40 %. Although this increase in performance seems to not be important, it is essential to point out that this is mainly due to the great complexity of the task undertaken. The resulting increase in performance obtained establishes that the HLDA and the voice conversion techniques can improve the discriminative properties of the cepstral frames used and therefore the recognition rates. So we think this article opens the way for further future successes in this very important topic that is the recognition of pathological voice.

## Conclusion and future works

In this paper, we present our hybrid system for improving the recognition of esophageal speech. This system is based on a simplified statistical GMM voice conversion that projects the esophageal frames into a clean laryngeal speech space. We do not use a speech synthesizer for reconstructing the converted speech signals, because the converted Mel cepstral vectors are used directly as input of the phone recognition system we used. We also projected the converted MFCC* vectors by the HLDA transformation into a smaller space for improving the discriminative properties. The obtained results demonstrate that our proposed hybrid system can improve the recognition of the esophageal speech. Concerning future works we are interested in realizing a portable device that will process the recognition of ES speech and synthesize the recognized speech using a text-to-speech synthesizer. Such a device would permit laryngectomees an easier oral communication with other people. However, the ES speech recognition system should be able to restore a greater part of the phonetic information (speech-to-text). For this reason, we intend to extend our FPSD corpus in order to make possible the use of context-dependent HMM models (triphones). Moreover, we plan to replace our simple voice conversion method by Toda’s algorithm [maximum likelihood estimation of spectral parameter trajectory considering global variance (GV) Toda et al. 2007] in order to improve the voice conversion process and consequently the accuracy of ES speech recognition.

## Footnotes

1 Hidden Markov Model.

2 Gaussian Mixture Model.

3 Represents the converted MFCC vectors.

4 http://www.praat.org.

5 http://www.phon.ucl.ac.uk/home/sampa/index.html.

6 Represents the converted logarithm energy.
